# CREST-2 and ECST-2: are we selecting the right patients?

**DOI:** 10.1590/1677-5449.202601332

**Published:** 2026-10-09

**Authors:** Marcone Lima Sobreira, Ricardo Augusto Carvalho Lujan

**Affiliations:** 1 Universidade Estadual Paulista – UNESP, Faculdade de Medicina de Botucatu, Botucatu, SP, Brasil.; 2 Universidade Federal da Bahia – UFBA, Hospital Universitário Professor Edgard Santos – HUPES, Salvador, BA, Brasil.

The publication of results from the Carotid Revascularization and Medical Management for Asymptomatic Carotid Stenosis (CREST-2) trials marked a milestone in the history of asymptomatic carotid stenosis treatment.^1^ In a setting in which medical therapy had evolved substantially since the landmark studies named Asymptomatic Carotid Atherosclerosis Study^2^ and Asymptomatic Carotid Surgery Trial,^3^ uncertainty remained regarding the role of prophylactic revascularization in patients without recent neurological symptoms.

The study findings were received enthusiastically by many specialists. Among patients with > 70% asymptomatic carotid stenosis, the addition of carotid artery stenting to intensive medical therapy reduced the incidence of the primary composite outcome in 4 years, whereas carotid endarterectomy showed no statistically significant benefit. At first glance, results suggest renewed interest in revascularization, particularly carotid artery stenting.

However, a closer reading suggests that this interpretation may be overly simplistic. Rather than providing a definite answer as to which therapeutic strategy should be adopted, CREST-2 appears to expose the limitations of the traditional model used to select patients for intervention.

Interestingly, the main finding of the study were not the performance of revascularization (surgical or endovascular), but the low incidence of events achieved with intensive medical therapy. Patients underwent a structured risk factor management program that included stringent blood pressure and cholesterol targets, systematic follow-up, behavioral counseling, encouragement of physical activity, smoking cessation, and access to PCSK9 inhibitors. This approach resulted in annual rates of ipsilateral stroke substantially lower than those observed in the landmark studies that formed the basis for current recommendations for revascularization in asymptomatic patients. This finding has immediate clinical relevance.

The natural history of asymptomatic carotid disease in 2026 is not the same as that observed in previous decades. Contemporary medical therapy has substantially reduced the absolute benefit of prophylactic revascularization. In other words, the lower a patient’s baseline risk, the more difficult it becomes to demonstrate a clinically meaningful benefit from any invasive procedure.

First, the magnitude of the observed absolute benefit should be considered. Although the stenting arm reached statistical significance, it is worth noting that, in 4 years, the incidence of the primary outcome was lower with stenting (2.8% vs. 6.0%), whereas no significant difference was observed between carotid endarterectomy and medical therapy (3.7% vs. 5.3%). These figures underscore both the low overall event rate and the relatively modest absolute benefit observed. In clinical terms, this represents a small difference compared with that observed in the landmark studies that established carotid endarterectomy as the standard of care for many asymptomatic patients.

Furthermore, the absolute number of events was low. When outcomes rates are low, even small variations in the number of events can substantially influence effect estimates, a limitation acknowledged by the authors themselves in the manuscript. This does not invalidate the findings but warrants caution when extrapolating the conclusions to broader populations.

Another aspect that deserves particular attention is the crossover rate observed in CREST-2. Approximately 18% of patients initially assigned to intensive medical therapy subsequently underwent revascularization (74% endovascular and 26% carotid endarterectomy). Although crossover tends to reduce differences between groups, it also points to an important practical limitation: the difficulty of maintaining a strictly conservative strategy in a substantial proportion of patients during follow-up. Rather than merely representing a methodological limitation, crossover serves as an indirect marker of risk heterogeneity that remains inadequately captured by traditional selection criteria. Ultimately, the fact that nearly 1 in 5 patients crossed over to an intervention suggests that the contemporary challenge lies not only in comparing therapeutic strategies but also in identifying early those patients whose clinical course may eventually lead to revascularization. This highlights that we may still be unable to adequately identify which patients will remain stable with medical therapy alone.

Moreover, the procedures were performed at highly specialized centers by previously certified operators and under rigorous quality control. Although this aspect strengthens the internal validity of a study, it inevitably limits its applicability to other settings. Outcomes achieved at centers of excellence may not always be replicated across heterogeneous health care systems or at lower-volume institutions.

Finally, the 4-year follow-up period should be interpreted in the context of a disease whose natural history often extends over decades. Differences observed within this time frame may change with longer-term follow-up.

These findings suggest that the interpretation of CREST-2 should extend beyond the simple dichotomy between medical therapy and revascularization. Although the study addressed important questions, it also left relevant questions unanswered for contemporary clinical practice (Table 1).

**Table 1:** What CREST-2 answered and what remained unanswered.

**CREST-2 answered**	**CREST-2 did not answer**
Contemporary intensive medical therapy substantially reduces stroke risk in asymptomatic patients	Which patients remain at high risk despite optimal medical therapy
The addition of carotid artery stenting to medical therapy reduced the incidence of the primary outcome at 4 years	Which characteristics identify patients who are most likely to benefit from intervention
Carotid endarterectomy showed no statistically significant benefit over the study period	Whether the benefit observed will remain unchanged or increase with longer-term follow-up
Procedures performed at specialized centers have low rates of periprocedural morbidity and mortality	Whether the results can be replicated in routine clinical practice
The current absolute stroke risk is lower than that observed in historical trials	What is the role of plaque vulnerability in treatment decisions
Revascularization remains an appropriate treatment option for selected patients	How to incorporate biomarkers, advanced imaging, and individualized risk stratification into patient selection for revascularization

CREST-2 = Carotid Revascularization and Medical Management for Asymptomatic Carotid Stenosis.

For decades, treatment decisions in carotid disease have relied primarily on symptom status and the degree of stenosis. However, patients with similar degrees of stenosis may have substantially different risks of stroke.

Perhaps the most important contribution of CREST-2 was to demonstrate that the degree of stenosis alone is insufficient to guide treatment decisions in asymptomatic patients. From this perspective, the study findings can be reinterpreted by clearly distinguishing the questions answered by CREST-2 from those that remain unanswered (Table 1).

The degree of stenosis is only an anatomical marker. Stroke risk depends on a far more complex interplay among plaque vulnerability, inflammatory activity, biological instability, subclinical embolization, cerebral hemodynamic reserve, and individual patient characteristics. Two patients with 80% stenosis may have entirely different prognoses.

The central question in contemporary practice may not be which treatment to choose – medical therapy, carotid endarterectomy, or carotid artery stenting – but rather which patients remain at risk despite the best available treatment. From this perspective, CREST-2 can be considered alongside other contemporary studies that have explored alternative approaches to patient selection and risk stratification.

The findings of these studies are also consistent with the 2023 Brazilian Guidelines for Carotid Artery Disease, which recommend an individualized approach to asymptomatic patients based not only on the degree of stenosis but also on life expectancy, perioperative risk, plaque characteristics, and the quality of medical therapy. From this perspective, the findings of CREST-2 and of the Second European Carotid Surgery Trial (ECST-2) appear to reinforce a paradigm shift already incorporated into national recommendations: rather than simply choosing between carotid endarterectomy and carotid artery stenting, the challenge is to identify which patients are truly likely to benefit from revascularization.^4^

In this context, the findings of ECST-2 are particularly relevant.^5^ Although CREST-2 and ECST-2 are often portrayed as competing studies, they may be better understood as complementary investigations. Whereas CREST-2 retained the degree of stenosis as the primary criterion for patient selection, ECST-2 sought to incorporate clinical risk models to identify patients at lower risk of future events. These methodological differences help explain both the apparent divergence in their conclusions and their convergence toward a similar overall message (Table 2).

**Table 2:** CREST-2 and ECST-2: methodological differences and clinical implications.

**Characteristic**	**CREST-2**	**ECST-2**	**Implication**
Population	Patients with ≥70% asymptomatic carotid stenosis	Low- and intermediate-risk asymptomatic and symptomatic patients	Different patient selection strategies
Primary inclusion criterion	Degree of stenosis	Estimated stroke risk (CAR score)	Anatomy vs. risk stratification
Comparison	CAS + BMT vs. BMT; CEA + BMT vs. BMT	Revascularization + BMT vs. BMT	Similar questions, different approaches
Medical therapy	Intensive and standardized	Optimal and contemporary	Representative of the contemporary landscape of vascular prevention
Main result	Statistically significant benefit with CAS; no statistically significant benefit with CEA	No statistically significant benefit of revascularization	The benefit of revascularization is not universal
Event rate	Very low	Very low	Lower contemporary baseline risk
Key message	Not all patients with ≥70% stenosis appear to have the same risk	Low- or intermediate risk may not require revascularization	Need for more refined patient selection
Question that remains	Which patients benefit from revascularization?	How can patients at higher risk of stroke be identified?	Future research priorities

BMT = best medical therapy; CAR = carotid artery risk; CAS = carotid artery stenting; CEA = carotid endarterectomy; CREST-2 = Carotid Revascularization and Medical Management for Asymptomatic Carotid Stenosis; ECST-2 = Second European Carotid Surgery Trial.

In contrast to CREST-2, the European study sought to select patients using risk prediction models that incorporated clinical characteristics in addition to the degree of stenosis. Preliminary findings showed no benefit from adding revascularization (carotid endarterectomy or carotid artery stenting) to optimal medical therapy in patients classified as being at low or intermediate risk. Rather than directly comparing the 2 studies, it may be more informative to recognize the convergence of their underlying messages. CREST-2 demonstrated the high effectiveness of contemporary medical therapy, whereas ECST-2 suggests that individualized risk stratification may be more relevant than the anatomical degree of stenosis alone.

Taken together, the 2 studies point to an important conceptual shift: identifying which patients are most likely to benefit from revascularization may be more important than determining which technique – carotid endarterectomy or carotid artery stenting – should be used.

One of the most apparent limitations of the contemporary literature on carotid disease is the persistence of a decision-making model overly focused on luminal anatomy. At the same time, a growing body of evidence links cerebrovascular events to specific atherosclerotic plaque features, including intraplaque hemorrhage, a large lipid-rich core, ulceration, inflammation, rapid lesion progression, and microembolization detected by transcranial Doppler. None of these features were central to patient selection in CREST-2. This should not be regarded as a methodological flaw of the trial. Rather, it highlights the gap between the criteria historically used in clinical trials and our growing understanding of the biology of carotid disease.

It is reasonable to expect that the next generation of studies will gradually move beyond a paradigm based solely on the degree of stenosis and symptom status, incorporating anatomical, functional, and biological markers to identify patients at higher risk of cerebrovascular events.

This shift in perspective reflects a broader transformation in our understanding of carotid disease. The landmark studies played a pivotal role in establishing the benefit of revascularization (carotid endarterectomy or carotid artery stenting) in an era of higher baseline risk and less effective medical therapy. Contemporary studies, by contrast, are conducted in a setting characterized by more effective, multifaceted medical therapy and growing interest in biomarkers of plaque vulnerability and individualized risk stratification (Table 3).

**Table 3:** What has changed from the era of landmark studies to the era of CREST-2 and ECST-2.

**The ACAS/ACST era**	**The CREST-2/ECST-2 era**
Decision-making based primarily on the degree of stenosis	Need for individualized risk stratification
Limited medical therapy	Highly effective medical therapy
Relatively high annual stroke risk	Very low annual stroke risk
More evident absolute benefit of revascularization	Smaller absolute benefit of revascularization, more difficult to demonstrate
Should patients with significant stenosis receive medical therapy?	Which patients remain at risk despite optimal medical therapy?

ACAS = Asymptomatic Carotid Atherosclerosis Study; ACST = Asymptomatic Carotid Surgery Trial; CREST-2 = Carotid Revascularization and Medical Management for Asymptomatic Carotid Stenosis; ECST-2 = Second European Carotid Surgery Trial.

CREST-2 does not appear to support an indiscriminate expansion of revascularization in asymptomatic patients. Rather, its key contribution, reinforced by ECST-2, was to demonstrate that contemporary medical therapy has substantially reduced stroke risk and that the current challenge is to identify patients who remain at residual risk and may benefit from revascularization. The focus of contemporary studies is therefore shifting from how to treat toward determining whom to treat.
