# Supplementary material for: CREST-2 and ECST-2: are we selecting the right patients?
Source: J Vasc Bras. 2026 Oct 9;25:e20260133. doi: 10.1590/1677-5449.202601332 (PMC13671005; doi:10.1590/1677-5449.202601332)
Supplement: Translation

# Translation: CREST-2 e ECST-2: estamos selecionando os pacientes certos?

**DOI:** 10.1590/1677-5449.202601331

**Authors:** Marcone Lima Sobreira, Ricardo Augusto Carvalho Lujan

**Affiliations:** 1 Universidade Estadual Paulista – UNESP, Faculdade de Medicina de Botucatu, Botucatu, SP, Brasil.; 2 Universidade Federal da Bahia – UFBA, Hospital Universitário Professor Edgard Santos – HUPES, Salvador, BA, Brasil.

A publicação dos resultados do *Carotid Revascularization and Medical Management for Asymptomatic Carotid Stenosis* (CREST-2) representou um marco na história do tratamento da estenose carotídea assintomática^1^. Em um cenário no qual o tratamento clínico evoluiu substancialmente desde os estudos clássicos *Asymptomatic Carotid Atherosclerosis Study* ^2^ e *Asymptomatic Carotid Surgery Trial* ^3^, permanecia a dúvida sobre o papel da revascularização cirúrgica profilática em pacientes sem sintomas neurológicos recentes.

Os resultados do estudo foram recebidos com entusiasmo por muitos especialistas. Em pacientes com estenose carotídea assintomática > 70%, a associação do stent carotídeo ao tratamento clínico intensivo reduziu a incidência do desfecho primário composto em 4 anos, enquanto a endarterectomia carotídea não demonstrou benefício estatisticamente significativo. À primeira vista, esses resultados sugerem uma valorização renovada da intervenção, especialmente do stent carotídeo.

Entretanto, uma leitura mais cuidadosa sugere que essa interpretação talvez seja simplista demais. Mais do que fornecer uma resposta definitiva sobre qual estratégia terapêutica deve ser adotada, o CREST-2 parece expor as limitações do modelo tradicional utilizado para selecionar pacientes candidatos à intervenção.

Curiosamente, o principal achado do estudo não foi o desempenho da revascularização (cirúrgica ou endovascular), mas a baixa incidência de eventos com o tratamento clínico intensivo. Os pacientes foram submetidos a um programa estruturado de controle dos fatores de risco, que incluía metas rigorosas de pressão arterial e colesterol, acompanhamento sistemático, aconselhamento comportamental, incentivo à atividade física, cessação do tabagismo e acesso a inibidores de PCSK9. Como resultado, as taxas anuais de acidente vascular cerebral (AVC) ipsilateral foram substancialmente inferiores às observadas nos estudos históricos que fundamentaram as recomendações atuais de revascularização em pacientes assintomáticos. Esse achado apresenta relevância clínica imediata.

A história natural da doença carotídea assintomática em 2026 não é a mesma observada em décadas passadas. O tratamento clínico contemporâneo reduziu substancialmente o benefício absoluto da intervenção profilática. Em outras palavras, quanto menor o risco basal do paciente, mais difícil se torna demonstrar um benefício clínico relevante de qualquer procedimento invasivo.

Embora o braço de stent tenha alcançado significância estatística, é importante destacar que, em 4 anos, a incidência do desfecho primário foi menor com o stent (2,8% vs. 6,0%), enquanto não houve diferença significativa entre a endarterectomia carotídea e o tratamento clínico (3,7% vs. 5,3%). Esses números refletem não apenas a baixa taxa global de eventos, mas também um benefício absoluto relativamente modesto. Em termos clínicos, trata-se de uma diferença pequena quando comparada àquela observada nos estudos clássicos que estabeleceram a endarterectomia carotídea como tratamento padrão para muitos pacientes assintomáticos.

Além disso, o número absoluto de eventos foi baixo. Em cenários de baixa incidência de desfechos, pequenas variações no número de eventos podem influenciar substancialmente as estimativas de efeito, uma limitação reconhecida pelos próprios autores no manuscrito. Isso não invalida os resultados, mas exige cautela ao extrapolar as conclusões para populações mais amplas.

Outro aspecto que merece atenção especial é a taxa de crossover observada no CREST-2. Aproximadamente 18% dos pacientes inicialmente alocados ao tratamento clínico intensivo foram posteriormente submetidos a revascularização (74% por via endovascular e 26% por endarterectomia carotídea). Embora esse fenômeno tenda a reduzir as diferenças entre os grupos, também revela uma importante limitação prática: a dificuldade de manter uma estratégia puramente conservadora em uma proporção significativa de pacientes durante o seguimento. Mais do que uma fragilidade metodológica, o *crossover* pode representar um marcador indireto de heterogeneidade de risco, ainda inadequadamente capturada pelos critérios tradicionais de seleção. Em última análise, o fato de quase um quinto dos pacientes ter migrado para uma intervenção sugere que o desafio contemporâneo não consiste apenas em comparar estratégias terapêuticas, mas também em identificar precocemente os indivíduos cuja evolução clínica poderá levar à revascularização. Isso evidencia que talvez ainda não sejamos capazes de identificar adequadamente quais pacientes permanecerão estáveis apenas com a tratamento clínico.

Ademais, os procedimentos foram realizados em centros altamente especializados, por operadores previamente certificados e sob rigoroso controle de qualidade. Embora esse aspecto fortaleça a validade interna do estudo, inevitavelmente limita sua aplicabilidade a outros contextos. Os resultados observados em centros de excelência nem sempre são reproduzidos em sistemas de saúde heterogêneos ou em instituições de menor volume.

Por fim, o seguimento de 4 anos deve ser interpretado no contexto de uma doença cuja história natural frequentemente se estende por décadas. É possível que as diferenças observadas nesse horizonte temporal se modifiquem com um seguimento mais prolongado.

Esses dados sugerem que a interpretação do CREST-2 deve ir além da simples dicotomia entre tratamento clínico e revascularização. Embora o estudo tenha respondido a questões importantes, também deixou questões relevantes em aberto para a prática clínica contemporânea (Tabela 1).

**Tabela 1:** O que o CREST-2 respondeu e o que permanece em aberto.

**O CREST-2 respondeu**	**O CREST-2 não respondeu**
O tratamento clínico intensivo contemporâneo reduz substancialmente o risco de AVC em pacientes assintomáticos	Quais pacientes permanecem em alto risco apesar do tratamento clínico otimizado
A angioplastia com implante de stent associada ao tratamento clínico reduziu a incidência do desfecho primário em 4 anos	Quais características identificam os pacientes que realmente se beneficiam da intervenção
A endarterectomia não demonstrou benefício estatisticamente significativo no período analisado	Se o benefício observado será mantido ou aumentará com um seguimento mais prolongado
Procedimentos realizados em centros especializados apresentam baixas taxas de morbimortalidade periprocedural	Se os resultados podem ser reproduzidos na prática clínica cotidiana
O risco absoluto de AVC atual é menor do que o observado nos estudos históricos	Qual o papel da vulnerabilidade da placa na tomada de decisão
A revascularização permanece uma opção terapêutica adequada para pacientes selecionados	Como incorporar biomarcadores, métodos avançados de imagem e estratificação individualizada do risco na seleção dos candidatos à intervenção

CREST-2 = *Carotid Revascularization and Medical Management for Asymptomatic Carotid Stenosis*.

Durante décadas, a decisão terapêutica na doença carotídea baseou-se na presença de sintomas e no grau de estenose. Entretanto, pacientes com graus semelhantes de estenose podem apresentar riscos muito diferentes de AVC.

O principal mérito do CREST-2 talvez tenha sido demonstrar que o grau de estenose, isoladamente, é insuficiente para orientar as decisões terapêuticas em pacientes assintomáticos. Sob essa perspectiva, é possível reinterpretar os resultados do estudo, distinguindo claramente as questões respondidas pelo CREST-2 daquelas que permanecem em aberto (Tabela 1).

A estenose é apenas um marcador anatômico. O risco de AVC depende de uma interação muito mais complexa entre a vulnerabilidade da placa, a atividade inflamatória, a instabilidade biológica, a embolização subclínica, a reserva hemodinâmica cerebral e as características individuais dos pacientes. Dois indivíduos com estenose de 80% podem apresentar prognósticos completamente distintos.

A principal questão contemporânea talvez não seja decidir entre tratamento clínico, endarterectomia carotídea ou stent. O desafio é identificar quais pacientes permanecem em risco apesar do melhor tratamento clínico disponível. Sob essa perspectiva, é interessante analisar o CREST-2 em conjunto com outros estudos contemporâneos que buscaram selecionar pacientes por meio de diferentes estratégias de estratificação de risco.

Os achados desses estudos também estão em consonância com as Diretrizes Brasileiras de Doença Carotídea de 2023, que recomendam uma abordagem individualizada para pacientes assintomáticos, baseada não apenas no grau de estenose, mas também na expectativa de vida, no risco perioperatório, nas características da placa e na qualidade do tratamento clínico. Sob essa perspectiva, os resultados do CREST-2 e do ECST-2 parecem reforçar uma mudança de paradigma já incorporada às recomendações nacionais: mais do que decidir entre endarterectomia carotídea e angioplastia carotídea com implante de stent, o desafio passa a ser identificar quais pacientes realmente se beneficiarão de alguma forma de revascularização^4^.

Dessa forma, os resultados do ECST-2 tornam-se particularmente relevantes^5^. Embora frequentemente apresentados como concorrentes, os estudos CREST-2 e ECST-2 provavelmente devem ser interpretados como investigações complementares. Enquanto o primeiro manteve o grau de estenose como principal critério de seleção, o segundo buscou incorporar modelos de risco clínico para identificar pacientes com menor probabilidade de eventos futuros. As diferenças metodológicas entre os dois estudos ajudam a compreender por que suas conclusões parecem divergir e, ao mesmo tempo, por que convergem para uma mensagem semelhante (Tabela 2).

**Tabela 2:** CREST-2 e ECST-2: diferenças metodológicas e implicações clínicas.

**Característica**	**CREST-2**	**ECST-2**	**Implicação**
População	Pacientes assintomáticos com estenose ≥ 70%	Pacientes assintomáticos e sintomáticos com risco baixo ou intermediário	Diferentes estratégias de seleção
Critério principal de inclusão	Grau de estenose	Risco estimado de AVC (escore CAR)	Anatomia *versus* estratificação de risco
Comparação	CAS + BMT vs. BMT; CEA + BMT vs. BMT	Revascularização + BMT vs. BMT	Questões semelhantes, abordagens diferentes
Tratamento clínico	Intensivo e padronizado	Otimizado e contemporâneo	Representativos do cenário contemporâneo da prevenção vascular
Resultado principal	Benefício com CAS; não significativo com CEA	Sem benefício da revascularização	Benefício da intervenção não é universal
Taxa de eventos	Muito baixa	Muito baixa	Menor risco basal contemporâneo
Mensagem principal	Nem todos os pacientes com estenose ≥70% parecem ter o mesmo risco	Pacientes de baixo/intermediário risco podem não necessitar de intervenção	Necessidade de seleção mais criteriosa dos pacientes
Questão que permanece	Quais pacientes se beneficiam da revascularização?	Como identificar os pacientes com maior risco de AVC?	Prioridades para pesquisas futuras

AVC = acidente vascular cerebral; BMT = melhor tratamento clínico (*best medical therapy*); CAR = risco da artéria carótida (*carotid artery risk);* CAS = angioplastia carotídea com implante de stent (*carotid artery stenting*); CEA = endarterectomia carotídea (*carotid endarterectomy*); CREST-2 = *Carotid Revascularization and Medical Management for Asymptomatic Carotid Stenosis;* ECST-2 = *Second European Carotid Surgery Trial*.

Diferentemente do CREST-2, o estudo europeu buscou selecionar pacientes com base em modelos preditivos de risco, incorporando características clínicas além do grau de estenose. Seus resultados preliminares não demonstraram benefício da revascularização (endarterectomia carotídea ou angioplastia carotídea com implante de stent), quando associada ao tratamento clínico otimizado, em pacientes classificados como de baixo ou intermediário risco. Mais importante do que comparar diretamente os dois estudos é reconhecer a convergência de suas mensagens. O CREST-2 demonstrou que o tratamento clínico contemporâneo é altamente eficaz. O ECST-2, por sua vez, sugere que a estratificação individualizada de risco pode ser mais relevante do que a simples quantificação anatômica da estenose.

Em conjunto, os dois estudos apontam para uma importante mudança conceitual: definir quais pacientes realmente se beneficiam de uma estratégia de revascularização tende a ser mais relevante do que decidir qual técnica – endarterectomia carotídea ou angioplastia carotídea com implante de stent – deve ser empregada.

Uma das limitações mais evidentes da literatura contemporânea sobre doença carotídea é a persistência de um modelo decisório excessivamente centrado na anatomia luminal. Paralelamente, acumulam-se evidências de uma associação entre eventos cerebrovasculares e características específicas da placa aterosclerótica, incluindo hemorragia intraplaca, núcleo lipídico volumoso, ulceração, inflamação, progressão acelerada da lesão e microembolização detectada por Doppler transcraniano. Nenhum desses elementos constitui um critério central para a seleção dos pacientes no CREST-2. Esse fato não representa uma falha metodológica do estudo. Na realidade, evidencia a distância entre os critérios historicamente utilizados nos ensaios clínicos e a crescente compreensão da biologia da doença carotídea.

É plausível supor que a próxima geração de estudos abandone progressivamente o paradigma baseado exclusivamente no grau de estenose e na presença de sintomas e passe a incorporar marcadores anatômicos, funcionais e biológicos capazes de identificar indivíduos verdadeiramente vulneráveis.

Essa mudança de perspectiva reflete uma transformação mais ampla na compreensão da doença carotídea. Os estudos clássicos foram fundamentais para demonstrar o benefício da estratégia de revascularização (endarterectomia carotídea ou angioplastia carotídea com implante de stent) em uma época caracterizada por maior risco basal e menor eficácia do tratamento clínico. Os estudos contemporâneos, por outro lado, inserem-se em um cenário marcado por um tratamento clínico mais efetivo (e multifatorial) e por crescente interesse em biomarcadores de vulnerabilidade da placa e estratificação individualizada de risco (Tabela 3).

**Tabela 3:** O que mudou entre a era dos estudos clássicos e a era do CREST-2 e do ECST-2.

**Era ACAS/ACST**	**Era CREST-2/ECST-2**
Decisão baseada principalmente no grau de estenose	Necessidade de estratificação individualizada de risco
Tratamento clínico menos eficaz	Tratamento clínico altamente eficaz
Risco anual de AVC relativamente elevado	Risco anual de AVC muito baixo
Benefício absoluto mais evidente da intervenção	Benefício absoluto menor e mais difícil de demonstrar
Quem apresenta estenose importante deve receber tratamento clínico?	Quem permanece em risco apesar do melhor tratamento clínico disponível?

ACAS = *Asymptomatic Carotid Atherosclerosis Study*; ACST = *Asymptomatic Carotid Surgery Trial*; AVC = acidente vascular cerebral; CREST-2 = *Carotid Revascularization and Medical Management for Asymptomatic Carotid Stenosis*; ECST-2 = *Second European Carotid Surgery Trial*.

O CREST-2 não parece justificar uma ampliação indiscriminada da revascularização em pacientes assintomáticos. Sua principal contribuição, reforçada pelo ECST-2, foi demonstrar que o tratamento clínico contemporâneo reduziu substancialmente o risco de AVC e que o desafio atual consiste em identificar quais pacientes permanecem sob risco residual e podem se beneficiar da revascularização. Mais do que definir como tratar, os estudos contemporâneos buscam determinar quais pacientes devem ser tratados.
